# Ectopic ACTH Secretion Secondary to Metastatic Acinic Cell Carcinoma of the Parotid Gland: A Case Report and Review of Current Evidence for Systemic Therapy

**DOI:** 10.1177/2324709620918080

**Published:** 2020-05-15

**Authors:** Louise Wade, Paul Kitching, Emma De Winton

**Affiliations:** 1 Royal United Hospitals Bath NHS Foundation Trust, Bath, UK

**Keywords:** salivary gland tumor, acinic cell carcinoma, ectopic adrenocorticotrophic hormone, ACTH

## Abstract

Acinic cell carcinoma is a rare, typically indolent, neoplasm that arises in the salivary glands. Metastatic disease is uncommon, occurring in around 10% of cases. We report the case of a 46-year-old male in whom the first sign of disseminated disease was increased skin pigmentation due to paraneoplastic Cushing’s syndrome. He underwent 3 cycles of chemotherapy with carboplatin and paclitaxel with no symptomatic improvement and a mixed response on imaging. There is no evidence that systemic therapy prolongs survival in metastatic acinic cell carcinoma, and we lack a consensus as to which treatment options are most beneficial. A summary of published evidence regarding choice of palliative chemotherapy regimens and response is discussed in relation to the case.

## Introduction

Acinic cell carcinoma (ACC) is a rare malignant tumor, arising almost exclusively in the salivary glands in which it accounts for 11% of malignant neoplasms.^1^ It is typically a low-grade, slow-growing tumor.^2^ Most patients present with localized disease, which is treated effectively with surgery and adjuvant radiotherapy, particularly if the disease was unable to be fully removed surgically.^2^ A minority of patients will go on to develop metastatic disease.^1^ At present, there is no consensus as to how to treat these patients and median survival is poor.^3^ We present an interesting case of metastatic ACC associated with paraneoplastic ectopic adrenocorticotrophic hormone (ACTH) secretion. This is followed by a review of the evidence that is currently available regarding palliative systemic therapy options for these patients.

## Case Report

This 46-year-old male underwent a superficial parotidectomy following presentation to our unit with a right parotid lump. He was previously fit and well with no family history of note. Histology confirmed a pT1 pN0 M0 ACC with risk factors including R1 resection at the deep margin and perineural invasion for which he completed adjuvant radiotherapy (60 Gy in 30 fractions) in February 2016. Post treatment magnetic resonance imaging (MRI) of his neck 3 months later showed no residual or recurrent disease, and he continued with standard 3 monthly clinical follow-up in the ENT (ear-nose-throat) clinic.

In April 2018, he complained of 3 episodes of headache, associated with temporary visual changes in his right eye, thought to be migraines. At follow-up 3 months later, he was noted to have tanned skin despite little sun exposure. Metabolic changes such as weight gain, hyperglycemia, or hypertension were not noted. He was referred via his general practitioner to a consultant gastroenterologist for investigation of hemochromatosis. By August 2018, he was reporting increased fatigue, had developed multiple subcutaneous nodules, and attended our emergency department with a focal seizure in his left arm, reported to follow another episode of migraine-type headache.

**Figure 1.**
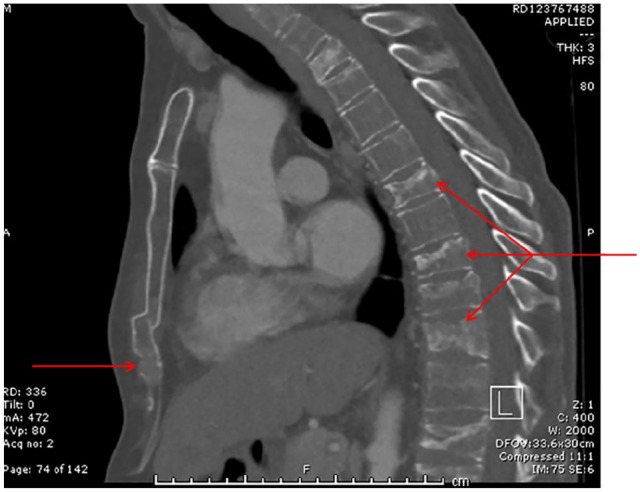
Sagittal section from the computed tomography (CT) scan of chest, abdomen, and pelvis taken in August 2018 demonstrating widespread boney metastatic deposits particularly affecting the sternum and vertebral bodies.

Imaging with computed tomography (CT) confirmed extensive disseminated malignancy with nodal, pleural, peritoneal, liver, and renal metastases (Figure 1). An MRI of head and whole spine showed metastases in the right frontal lobe and bilateral parietal lobes, a deposit along the L4 nerve root, as well as bone metastases throughout his cervical, thoracic, and lumbar spine. There was no evidence of locoregional relapse.

**Figure 2.**
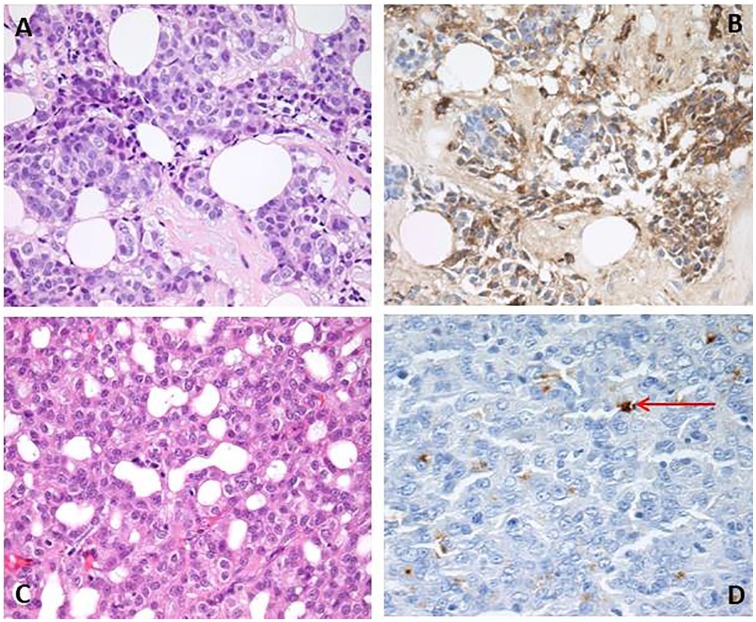
Photomicrographs “A” and “B” are from the supraclavicular fossa nodal biopsy. “A” shows poorly differentiated carcinoma. “B” shows staining for α1-antichymotrypsin. Photomicrographs “C” and “D” are from his original parotidectomy specimen. “C” shows appearances of acinic cell carcinoma. “D” demonstrates immunostaining with focal expression of ACTH (as indicated by the red arrow).

Biopsy from a supraclavicular fossa node showed poorly differentiated carcinoma infiltrating within fat (Figure 2). Immunostaining for α1-antichymotrypsin, a marker commonly expressed in ACC, was positive. Review of the previous histology from the right parotidectomy showed an ACC, mostly showing a microcystic pattern but with more poorly differentiated areas with higher proliferation, resembling the metastatic lesion from 2018. Again, expression of α1-antichymotrypsin was demonstrated on immunostaining. It was concluded that the supraclavicular fossa lesion represented metastatic ACC. Subsequent immunostaining of the original tumor showed focal expression of ACTH within scattered tumor cells, consistent with the clinical picture of increased skin pigmentation. The nodal biopsy was too small to perform ACTH staining on. No genetic studies were done on the tumor.

On examination, he had Cushingoid fat distribution, hyperpigmentation, signs of agitation, proximal weakness, and hypokalemia at 2.5 mmol/L. An early morning cortisol level was markedly high at 1619 nmol/L in keeping with Cushing’s syndrome. In view of the very high cortisol level and significant symptoms, our endocrinology team did not want to delay starting treatment. Consequently, other tests, such as a dexamethasone suppression test, were not performed at this time. He was started on a block and replace regimen of metyrapone and dexamethasone the same day, with a view switching to hydrocortisone pending response to chemotherapy. The MRI of head had shown unremarkable appearances of the anterior and posterior pituitary. Ectopic ACTH secretion was the likely underlying cause.

He commenced palliative chemotherapy with carboplatin and paclitaxel. In addition, he had a single 8 Gy fraction of palliative radiotherapy to his lumbar spine for pain.

Despite chemotherapy, he deteriorated with increasing generalized edema, back pain, and restrictions in mobility along with development of distal weakness in his right upper limb. His cortisol levels improved to 558 nmol/L, and his potassium normalized at 3.7 mmol/L. Repeat imaging after 3 cycles confirmed progressive disease in his liver and extensively throughout the spine along with a new metastatic deposit on the left adrenal gland. His nodal and intracranial disease was stable, and there was some regression in the right renal metastasis. By this point, his performance status had fallen to World Health Organization 3, and a decision was made with the patient to stop chemotherapy and focus on symptomatic care.

## Discussion

A literature search revealed a number of published cases of metastatic ACC with ectopic ACTH secretion.^4-10^ In 4 cases, chemotherapy treatment was given with varying responses. The best documented outcome was of a 15-month survival from presentation with metastatic disease.^7^ Ectopic ACTH secretion is associated with a poorer prognosis regardless of primary site.^11^

Metastatic disease secondary to ACC is uncommon, occurring in 10% of patients with late metastatic relapse recognized.^3^ There is no good evidence that systemic treatment of metastatic disease prolongs survival, and there is no standard treatment regimen.^3^ Platinum-based chemotherapy, either as monotherapy or in combination, is the most commonly described approach.^3^ The response rate to CAP (cyclophosphamide, doxorubicin, and cisplatin) is reported as 46%, but this data refers to all histological subtypes of salivary gland tumors of which ACCs only form a small portion.^3^ There is no phase III data for chemotherapy in this group of patients.^12^ Table 1 comprises a literature review of published evidence on responses to first-line palliative chemotherapy in metastatic ACC.

**Table 1.** Summary of Published Evidence of Response to First-Line Palliative Chemotherapy in Metastatic ACC.

Author	Year	Type of Study	No. of Patients Included With ACC	Of Patients With ACC, No. of Which Also Had Ectopic ACTH Secretion	Regimen (Doses Included Where Available)	Response
Oliveira et al^7^	2019	Case report	1	1	Weekly carboplatin and paclitaxel, on PD second-line oral vinorelbine	Survived for 15 months after diagnosis of metastatic disease
Saluja et al^8^	2019	Case report	1	1	6 cycles of carboplatin and paclitaxel	PR, TTP 4 months after completing primary chemotherapy
Khelfa et al^3^	2016	Case report	1	0	6 cycles of carboplatin and paclitaxel followed by single-agent paclitaxel	Significant PR, TTP 8 months
De Block et al^13^	2016	Case series (of salivary gland tumors)	1	0	6 cycles of cyclophosphamide, doxorubicin, and cisplatin	CR, TTP 19 months
Neren et al^14^	2015	Case report	1	0	Cisplatin and cetuximab, given 3 weekly for 2 years	24 months after presentation SD
Debaere et al^15^	2011	Retrospective review (of a series of 15 cases)	1	0	6× cyclophosphamide (600 mg/m^2^), doxorubicin (50 mg/m^2^), and cisplatin (50 mg/m^2^), 3 weekly (required a DR from cycle 2 onward)	PR, TTP 383 days
Shenoy et al^4^	2011	Case report	1	1	1× doxorubicin (50 mg/m^2^)	Died after 1 cycle due to neutropenic sepsis
Butt et al^10^	2008	Case report	1	1	Gemcitabine and docetaxel	Clinical and biochemical response after 2 cycles
Vidyadhara et al^16^	2007	Case report	1	0	9× cisplatin, epirubicin, 5-flurouracil	Initial improvement in symptoms, TTP 6 months
Creagan et al^17^	1988	Case series (of salivary gland tumors)	1	0	Cyclophosphamide 200 mg/m^2^, doxorubicin 30 mg/m^2^, cisplatin 90 mg/m^2^ 24-hour continuous infusion, given monthly	PR after 6 cycles, stopped treatment after 16 cycles for toxicity, TTP 4.9 years
Posner et al^18^	1982	Case series	3	0	Cyclophosphamide 450 mg/m^2^, adriamycin 45 mg/m^2^	1 PR, with survival of >12 months (Note: this patient had 3 cycles of chemotherapy and then proceeded with a second surgical excision of residual disease and adjuvant radiotherapy)

Abbreviations: ACC, acinic cell carcinoma; PD, progressive disease; PR, partial response; TTP, time to progression; CR, complete response; SD, stable disease; DR, dose reduction.

The phase 1b Keynote-028 study looked at the anti-PD-1 monoclonal antibody pembrolizumab in advanced salivary gland carcinoma, and 1 out of 26 enrolled patients had ACC.^19^ This patient had a documented reduction in tumor size but did not reach the criteria for a partial response. The median duration of response for all participants in the study was 3.9 months.

Little is known about the genetic changes present in ACC.^20^ Genetic alterations in 25 cases of primary parotid ACC were studied by El-Naggar et al.^21^ The dominant alteration found was loss of heterozygosity indicating the potential role of tumor suppressor genes. A more recent study used mice models to show the effect of deleting 2 tumor suppressor genes—adenomatous polyposis coli and phosphatase and tensin homologue. This caused activation of the mTOR pathway and led to formation of salivary gland tumors, morphologically similar to ACC, with 100% penetrance.^22^ Treatment of the tumor-bearing mice with rapamycin, an mTOR inhibitor, lead to complete regression of tumors. This may prove to be a useful clinical target in the future.

In summary, we report a case of a young man with metastatic ACC and ectopic ACTH production. Despite no chemotherapy toxicity and a mixed response to treatment, he did not benefit symptomatically from palliative chemotherapy and deteriorated over a short period before active treatment was withdrawn. The diagnosis of paraneoplastic syndrome causing ectopic ACTH production in patients presenting with a skin pigment change should be within the differential, particularly in the context of prior cancer treatment.
